# Discharge of surplus phloem water may be required for normal grape ripening

**DOI:** 10.1093/jxb/erw476

**Published:** 2017-01-12

**Authors:** Yun Zhang, Markus Keller

**Affiliations:** 1 Irrigated Agriculture Research and Extension Center, Washington State University, 24106 N. Bunn Road, Prosser, WA 99350, USA

**Keywords:** Fruit ripening, fruit water relations, grape berry, hydrostatic pressure, phloem unloading, transpiration, *Vitis*, xylem flow

## Abstract

At the onset of ripening, some fleshy fruits shift the dominant water import pathway from the xylem to the phloem, but the cause for the decline in xylem inflow remains obscure. This study found that xylem-mobile dye movement into grape berries decreased despite transient increases in berry growth and transpiration during early ripening, whereas outward dye movement continued unless the roots were pressurized. Modeling berry vascular flows using measurements of pedicel phloem sap sugar concentration, berry growth, solute accumulation, and transpiration showed that a fraction of phloem-derived water was used for berry growth and transpiration; the surplus was recirculated via the xylem. Changing phloem sap sugar concentration to a much higher published value led to model simulations predicting xylem inflow or backflow depending on the developmental stage and genotype. Mathematically preventing net xylem flow resulted in large variations in phloem sap sugar concentration in pedicels serving neighboring berries on the same fruit cluster. Moreover, restricting water discharge via the xylem and/or across the skin impaired berry solute accumulation and color change. Collectively, these results indicate that discharge of surplus phloem water via berry transpiration and/or xylem backflow may be necessary to facilitate normal grape ripening.

## Highlight

Developmental changes in xylem flow at the onset of grape ripening may be associated with a surplus of phloem-derived water. Discharge via fruit transpiration and xylem backflow facilitates normal ripening.

## Introduction

Fleshy fruits are terminal sinks that rely on water and solute import via the plant’s vascular system (Johnson *et al.*, 1992; Matthews and Shackel, 2005). Water is imported via both the xylem and the phloem, while sugars (the major solutes in a ripe fruit) are imported by the phloem. Fruit water content is an important determinant of fruit quality, because water serves as a solvent for and determines the concentration of sugars, acids, phenolics, and other organic and inorganic compounds (Coombe and Iland, 2004; Keller, 2015). In addition, water content affects fruit turgor, decrease in which is a suspected trigger for the onset of ripening (Kawabata *et al.*, 2002; Matthews *et al.*, 2009). Imbalances of water content may cause fruit shrinkage (insufficient water) or splitting (too much water). Thus, an understanding of fruit water relations is paramount for the development of strategies aimed at optimizing fruit quality.

Similar to other fleshy fruits, the dominant water import pathway for grape (*Vitis* sp.) berries shifts from the xylem to the phloem at the onset of ripening (termed veraison), and the amount of water entering berries via the xylem decreases (Matthews and Shackel, 2005; Keller *et al.*, 2015). However, the berry xylem remains physically intact and without occlusion throughout ripening (Chatelet *et al.*, 2008*a*, *b*; Choat *et al.*, 2009). Although deposition of polysaccharide-like material has been reported in the pedicel xylem vessels, this was observed only after veraison, and not around the onset of ripening (Knipfer *et al.*, 2015). Therefore, the decline in xylem inflow at veraison cannot be explained by a loss of xylem functionality.

The rate of water transport through xylem vessels can be stated as the product of xylem hydraulic conductance (*k*_h_) and the hydrostatic pressure gradient (∆*P*_x_) in the xylem (Nobel, 2009). Pedicel and berry *k*_h_ did not change at the onset of ripening (Choat *et al.*, 2009; Knipfer *et al.*, 2015). Evidently, the sudden developmental decline in xylem inflow into the berries is not due to changes in *k*_h_. Recent research from our laboratory suggests that this decline may instead be a consequence of the sink-driven increase in phloem inflow at the beginning of ripening, leading to a change in the predominant direction of the ∆*P*_x_ (Keller *et al.*, 2015). If the direction of ∆*P*_x_ is from the fruit to the leaves, fruit may experience reversed xylem flow (also known as xylem backflow), which has been reported in various species (Choat *et al.*, 2009; Clearwater *et al.*, 2012; Higuchi and Sakuratani, 2006; Johnson *et al.*, 1992; Keller *et al.*, 2006; Lang and Thorpe, 1989; Nobel *et al.*, 1994; Tilbrook and Tyerman, 2009). Similar to tomato (*Solanum lycopersicum* L.) fruit, the shift in the major water import pathway in grape berries coincides with the switch of phloem unloading from a symplastic to an apoplastic pathway (Ruan and Patrick, 1995; Zhang *et al.*, 2006). The resulting presence of solutes in the fruit apoplast (Keller and Shrestha, 2014; Ruan *et al.*, 1996) increases the osmotic pressure of the apoplast (*π*_a_) (Keller *et al.*, 2015; Wada *et al.*, 2008, 2009). This increase in *π*_a_ enhances osmotic water outflow from the phloem, thus releasing the sink phloem pressure (Patrick, 1997). It is possible that water outflow from the phloem increases the fruit apoplast pressure (*P*_a_) and thus alters Δ*P*_x_ (Keller *et al.*, 2015). Additionally, ∆*P*_x_ may be altered by developmental changes in berry transpiration rate (*E*), which is thought to decrease during ripening (Becker and Knoche, 2011; Rogiers *et al.*, 2004).

In this study, genetically distinct grape cultivars were used to further our understanding of the developmental changes in vascular flows in fleshy fruits at the onset of ripening. Direct measurements of vascular flows have been reported for peduncles of mango fruit (*Mangifera indica* L.; Higuchi and Sakuratani, 2006) and tomato truss (Windt *et al.*, 2009), and pedicels of kiwifruit berries (*Actinidia chinensis* Planch.; Clearwater *et al.*, 2012). However, the small size of grape berry pedicels (~1 mm in diameter, ≤1 cm in length) currently limits measurements using either miniature sap flow sensors (diameter ≥2 mm; Clearwater *et al.*, 2009) or nuclear magnetic resonance flow imaging (diameter ≥3 mm; Windt *et al.*, 2011). In addition, the outer tip diameter of the cell pressure probe (4–100 μm; Wei *et al.*, 2001; Wong *et al.*, 2009) exceeds the average diameter of xylem conduits in grape berries (3–5 μm; Chatelet *et al.*, 2008*b*). Because these technical limitations impede direct measurements in grapes, we conducted a series of indirect experiments, starting with dye tracer observations and measurements of fruit transpiration. We also measured pedicel phloem sap sugar concentration and estimated vascular flow rates using a fruit growth model. Finally, we conducted hydraulic manipulations and quantified their impacts on berry ripening.

## Materials and methods

### Plant material

Own-rooted grapevines *Vitis vinifera* L., cvs. Merlot and Syrah (planted in 1999), and *Vitis labruscana* Bailey, cv. Concord (planted in 2003), were used for field experiments and sample collection from 2009 to 2011. These vines were grown in the experimental vineyards of the Irrigated Agriculture Research and Extension Center in Prosser, WA, USA (46°17ʹ N; 119°44ʹ W; elevation 365 m; mean annual and growing season precipitation 171 mm and 92 mm, respectively, over the study years). The vines were drip-irrigated and grown at a planting distance of 1.8 m by 2.7 m in north–south-oriented rows down a ≤2% south-facing slope.

Two-year-old, own-rooted Merlot, Syrah, and Concord grapevines were grown in white 20 l PVC pots containing a mixture of 50% sandy loam, 25% peat moss, 25% pumice, and 30 g l^–1^ dolomite. These vines were grown outside and moved into an air-conditioned greenhouse for each experiment. Supplemental light maintained a minimum photoperiod of 12 h (temperature 18–25 °C) and midday photosynthetically active radiation >1000 μmol photons m^–2^ s^–1^. The pots were irrigated daily, and 5 g of Mora-Leaf^®^ Plus fertilizer (Wilbur-Ellis, Kennewick, WA, USA) was applied to each pot before anthesis.

### Berry maturity

Data were collected from individual berries. The berries were grouped into successive developmental stages based on their firmness to the touch and skin color (Keller and Shrestha, 2014; Keller *et al.*, 2015; Zhang and Keller, 2015), coded as follows: green hard=1, green soft=2, blush/pink=3, red/purple=4, and blue=5. After the berries turned blue, two further groups were categorized according to total soluble solids (TSS): ripe=6 (Merlot and Syrah: 20–24 °Brix; Concord: 18–20 °Brix) and overripe=7 (Merlot and Syrah: >24 °Brix; Concord: >20 °Brix). Berry fresh weight (FW) and TSS (measured by handheld refractometer, Atago, Tokyo, Japan) were determined for each stage. Solute content per berry (*S*) was estimated from FW and TSS, and osmotic pressure (*π*, in MPa) was estimated from TSS. Preliminary measurements with Merlot and Concord berries had shown that TSS was tightly correlated with *π* calculated from osmolality as measured using a vapor pressure osmometer (Vapro 5520; Wescor, Logan, UT, USA; see also Keller *et al.*, 2015). For the measured range of 5.8–25.2 °Brix we found a common quadratic regression equation for the two genotypes: (*π*=0.383 + 0.102×TSS+0.004×TSS^2^; *r*>0.99, *P*<0.001, *n*=127; see Supplementary Fig. S1 at *JXB* online). Strong correlations were obtained between TSS (and *π*) and developmental stages (codes) in all three genotypes (*r*>0.95, *P*<0.001, *n*=50). All statistical analyses were conducted in Statistica 12 (StatSoft, Tulsa, OK, USA).

### Solute concentration in berry sections

Fruit clusters of Merlot, Syrah, and Concord were collected from the experimental vineyard immediately before and throughout ripening. Individually, 104 Merlot, 114 Syrah, and 55 Concord berries were cut into three sections, named distal end, middle, and proximal end (Supplementary Fig. S2). The two cuts were adjacent to the seeds. TSS was measured separately for each section. The association between TSS in the distal and proximal end sections was tested by correlation analysis. The difference in TSS between these two sections (ΔTSS) was analyzed by paired *t* test.

### Berry transpiration and dye infusion to detached clusters

Shoots with fruit clusters of Merlot, Syrah, and Concord were collected as mentioned above. Immediately after cutting the shoots off the vines, their cut ends were immersed and recut under water, and they were transferred to the laboratory. Individual berries (*n*>50 for each genotype) from one set of these clusters were used to estimate *E* per berry by weighing them over time as described by Zhang and Keller (2015). Afterwards, TSS of individual berries was measured. Another set of clusters was used to quantify the mobility of xylem-mobile dye into berries. The cut end of the peduncle was immediately immersed in a centrifuge tube with 30 ml of a 0.1% (w/v) aqueous solution of basic fuchsin (Sigma-Aldrich, St. Louis, MO, USA), and the tube was sealed and placed on the laboratory bench for 24 h. Berries (*n*>50 for each genotype) were sectioned longitudinally to observe dye movement through the berry vasculature. The location of the dye in the berry was rated visually and assigned a number from 1 (dye in the brush or proximal end only) to 6 (dye throughout the entire vascular network) (Keller *et al.*, 2006). Then, TSS was measured for each berry. The correlations between TSS and *E*, as well as between TSS and dye location, were analyzed, and curves were fitted using the Lowess method.

### Reverse dye infusion with and without external pressure

Previously, we found that basic fuchsin fed to the distal end of attached grape berries moved spontaneously back to the shoot (Keller *et al.*, 2006). In order to test whether this outward dye movement could be restricted by external pressure, the root system of potted Merlot and Concord vines was pressurized in a custom-built 26 l root pressure chamber (Keller *et al.*, 2006, 2015). The gas pressure applied to the roots was increased at ~0.01 MPa min^–1^ to the point at which sap exuded from cut pedicels. Exudation occurred at between 0.1 and 0.3 MPa and was taken as evidence that the pressure was at or above the balancing pressure required to keep the plants at full turgor (Dodd *et al.*, 2002; Passioura, 1988). The purpose of root pressurization was simply to test whether or not applied pressure would restrict outward dye movement, rather than to determine the exact value of the balancing pressure. Once exudation occurred, the pressure was maintained, and the distal end of one berry per cluster was quickly removed with a fresh razor blade to expose the vascular bundles. Cutting is unlikely to have altered *P*_x_ significantly, since *P*_x_≈0 in ripening grape berries (Bondada *et al.*, 2005; Tyerman *et al.*, 2004). The cut end of the berries was immediately immersed in basic fuchsin solution (Keller *et al.*, 2006). For one set of plants, the pressure was then released, and for the other set the pressure was maintained. After 2 h, berries were removed from the vines, and cross-sections of berries and their pedicels were observed using an AxioCam ERc5s camera attached to a Zeiss SteREO Discovery.V12 (Carl Zeiss Microscopy, Göttingen, Germany). The TSS was measured in the removed distal end of each berry. Four to six berries per vine of two vines per cultivar were tested for each condition.

### Pedicel phloem sap collection and sap sugar concentration

In order to estimate phloem sap sugar concentration near the release phloem, phloem sap was collected in the greenhouse from pedicels of Merlot, Syrah, and Concord berries ranging from green hard to blue (*n*≥5). Sap was collected between 8.00 and 12.00 h local standard time. The developmental stage, FW, and TSS of berries from sampled pedicels were recorded. Because sap-sucking insects do not feed on grape pedicels, which precludes the use of stylectomy for phloem sap collection, we modified the classic phloem exudation technique that uses low concentrations of EDTA (2-Na-ethylenediamine tetraacetic acid; Sigma-Aldrich, St. Louis, MO, USA) to prevent phloem callose formation while minimizing cell damage and hence leakage from adjacent cells (Najla *et al.*, 2010; van Bel and Hess, 2008). Two collection solutions were prepared: ‘EDTA’ as 5 mM EDTA in 10 mM HEPES [4-(2-hydroxyethyl)-1-piperazineethanesulfonic acid; Sigma-Aldrich, St. Louis, MO, USA] buffer, and ‘HEPES’ as 10 mM HEPES buffer adjusted to pH 7.5 with KOH. Collection tubes were made from 200 μl plastic HPLC vial inserts (Micro SOLV, Eatontown, NJ, USA), their initial weights were recorded, and 80 μl of collection solution was added. Using a fresh razor blade dipped in ‘EDTA’, a berry was cut from the end of its pedicel. The cut on the plant side was immediately rinsed with ‘EDTA’ and immersed in the ‘EDTA’ collection tube. The tube was quickly attached to the pedicel with Parafilm® in order to avoid evaporation, wrapped in aluminum foil to exclude sunlight, and removed after 1 h. The pedicel was quickly rinsed with ‘HEPES’ to remove any remaining EDTA, and immersed in the ‘HEPES’ tube for 1 h. Both tubes were weighed immediately upon removal and stored at –80 °C.

Sugars (sucrose, glucose, and fructose) in both collection solutions were measured by HPLC using an Agilent 1100 system (Agilent Technologies, Santa Clara, CA, USA) as described by Keller and Shrestha (2014). When hexoses were detected in any ‘HEPES’ tubes, the corresponding ‘EDTA’ tubes were assumed to be contaminated with cell sap and discarded. The amount of sucrose in the phloem sap was calculated from the ‘EDTA’ volume in the collection tube and the measured sucrose concentration. The changes in the weight of ‘EDTA’ tubes reflected both phloem sap exudate and xylem sap uptake, while the changes in ‘HEPES’ tubes reflected only xylem sap uptake (Najla *et al.*, 2010). It was assumed that the rate of xylem sap uptake was constant during the 2 h collection period. Therefore, the amount of phloem sap was estimated as the difference in weights of the two tubes, and the volume of phloem sap was calculated, assuming a density of 1 g ml^–1^. The sucrose concentration (*C*_p_) was calculated from the volume of phloem sap and the amount of sucrose. Two-way ANOVA was used to evaluate the variation in *C*_*p*_ due to genotypes and developmental stages. Due to the lack of significance among genotypes (*P*=0.31), values of *C*_*p*_ were pooled for all three genotypes, and one-way ANOVA was conducted to evaluate differences in *C*_p_ during development.

### Model of fruit growth to quantify vascular flows

Abbreviations and units of measurements used in the model are listed in Table 1. Grape berry FW without seeds (*M*) of Merlot, Syrah, and Concord at varying developmental stages was calculated by subtracting seed weight from FW (seed weight=0.05×FW; *n*=83, *r*=0.90, *P*<0.001). We made the simplifying assumption that *M*=*W*+*S*, where *W* is berry water content and *S* is solute content. Additionally, *S* was estimated as the product of TSS and FW.

**Table 1.** Symbols, abbreviations and units of measurement used for the fruit growth model

Abbreviation	Definition	Unit
*M*	Grape berry fresh weight without seeds	g
*W*	Grape berry water content	g
*S*	Grape berry solute content	g
TSS	Total soluble solids	°Brix
d*W* (d*t*)^−1^	Daily change in berry water content	g d^−1^
*W*_xi_	Daily water inflow via the xylem	g d^−1^
*W*_xb_	Daily water backflow via the xylem	g d^−1^
Δ*W*_x_	Daily net xylem flow	g d^−1^
*W*_p_	Daily water inflow via the phloem	g d^−1^
*E*	Daily berry transpiration	g d^−1^
d*S* (d*t*)^−1^	Daily change in berry solute content	g d^−1^
*S*_p_	Daily sugar inflow via the phloem	g d^−1^
*R*	Daily solute consumption by respiration	g d^−1^
*C*_p_	Sucrose concentration in the pedicel phloem	mM
*C*_p_’	*C*_p_ for Δ*W*_x_=0	mM
*V*_p_	Daily volume of phloem inflow	l d^−1^

The changes in *W* can be described as:

$$\text{d}W{\left(\text{d}t\right)}^{–\text{1}}=W_{\text{xi}}+W_{\text{p}}–W_{\text{xb}}–E=\Delta W_{\text{x}}+W_{\text{p}}–E$$ (1)

where d*W* (d*t*)^–1^ is the daily change in *W*; *W*_xi_ and *W*_p_ are the daily water inflows via the xylem and the phloem, respectively; *W*_xb_ and *E* are the daily water outflows via xylem backflow and berry transpiration, respectively; and Δ*W*_x_ is the daily net xylem flow. Positive values for Δ*W*_x_ indicate xylem inflow, and negative values indicate xylem backflow.

Because grape berries accumulate nearly no starch (Amerine and Bailey, 1959) and their photosynthetic rate is negligible (Blanke and Lenz, 1989), the phloem is virtually their only supply of sugars. Moreover, solutes accumulated by ripening berries are dominated by sugars (Keller and Shrestha, 2014). Therefore, the changes in *S* can be described as:

$$\text{d}S{\left(\text{d}t\right)}^{–\text{1}}=S_{\text{p}}–R=C_{\text{p}}\times V_{\text{p}}–R=C_{\text{p}}\times (W_{\text{p}}/\text{1}000)–R$$ (2)

where d*S* (d*t*)^–1^ is the daily change in *S*; *S*_p_ is the daily sugar inflow via the phloem; *R* is the daily consumption of sugar through respiration; *V*_p_ is the daily volume of phloem inflow; and 1000 is a factor used for unit conversion. Phloem sap density was assumed to be 1 g ml^–1^ due to the low *C*_p_ (see Results).

The objects of this model are individual berries rather than whole clusters. On average, once a berry reached the green soft stage, it took ~3 d to reach each successive stage until it reached the blue stage as defined above. Then, it generally took ~7 d for a berry to reach the ripe stage, and an additional ~14 d to reach the overripe stage. For simplicity, the intervals between successive developmental stages were taken to be the same for the three genotypes. With this basis, *W*_p_ was obtained from equation 2, using measured *S*, measured *C*_*p*_, and published *R* values (Keller *et al.*, 2015; assuming the same *R* for Syrah and Merlot). Then, Δ*W*_x_ was calculated according to equation 1. In addition, the average *C*_p_ (18.2% w/w=532 mM) of transport phloem across diverse species reported by Jensen *et al.* (2013) was used to calculate *W*_p_ and Δ*W*_x_. Finally, for the hypothetical scenario that there was no net xylem flow between a berry and its pedicel, or Δ*W*_x_=0 (i.e. *W*_p_=*E*+d*W* (d*t*)^–1^), values of *C*_p_ were calculated and denoted *C*_p_’. Effects of genotypes and developmental stages on *W*_p_, Δ*W*_x_, and *C*_p_’ were analyzed using two-way ANOVA. Due to the significant interaction between genotypes and stages, one-way ANOVA was then conducted within genotypes, and differences due to development were evaluated using Fisher’s least significant difference test.

### Manipulation of xylem flow and berry transpiration

To answer the question whether restricting one or both of the two possible water outflow pathways from grape berries (i.e. berry transpiration and xylem backflow) could alter phloem inflow into the berries, 10 vines each of Merlot, Syrah, and Concord were randomly selected in the experimental vineyard. To minimize variations in the microclimate for berry ripening, four fruit clusters that were adjacent to one another, on the eastern side of the canopy, and not shaded by foliage were tagged on each vine. One each of the four clusters per vine was randomly assigned to one of three treatments or to serve as the control. The treatments were: restricted xylem flow (-X), restricted berry transpiration (-T), and the combination of both (-XT). Xylem tissue was destroyed for -X and -XT by drilling as described elsewhere (Hall *et al.*, 2011; Keller *et al.*, 2015). To minimize damage to the phloem, drilling was conducted very carefully, and clusters that developed necrotic peduncles were discarded. Berry transpiration was restricted for -T and -XT by applying a commercial antitranspirant (Vapor-Gard; Miller, Hanover, PA, USA), which reduced transpiration by ~30% (Zhang and Keller, 2015). Each cluster was immersed three times in a 2% antitranspirant solution to ensure complete coverage (S. Poni; personal communication).

Treatments were applied on two occasions: just before veraison, when all berries were green hard (early ripening treatment), and again using a different set of clusters with only blue-colored berries (late ripening treatment). Five berries from each cluster were randomly sampled before and 14 d after treatment application to record FW and TSS. The absolute rate of volumetric berry growth was estimated from the change in FW and density as estimated from TSS (Keller *et al.*, 2015). Berry *S* was estimated as mentioned above to calculate the absolute rate of solute accumulation. As berry splitting was observed in some cases, the number of split berries and the total number of berries on each cluster were recorded. Factorial ANOVA was conducted to test treatment effects on berry development and splitting frequency. Due to significant interaction between treatment time and genotype, one-way ANOVA was then conducted within genotypes at each treatment time, and differences among treatments were evaluated using Fisher’s least significant difference test.

An additional, independent experiment was conducted in which root pressurization was applied during berry ripening. The root system of potted Syrah vines with berries ranging from green hard to blue was pressurized at 0.3 MPa for 3 d. This pressure was used because it was found to be adequate to stop the outward movement of xylem-mobile dye from the berries (see results of reverse dye infusion experiment). Berry development on pressurized vines was compared with that on unpressurized (control) vines. Five pairs of berries at each developmental stage were tagged before the experiment. The berries of each pair had similar diameters (measured with digital calipers) and were next to each other. One berry of each pair was sampled before pressurization to record the initial FW and TSS; the other berry was sampled immediately after pressurization was stopped. Berry *S* was calculated as described above. To test whether or not the applied pressure altered leaf photosynthesis (i.e. source activity), leaf gas exchange was measured on fully expanded leaves (*n*=10) between 9.00 and 12.00 h local standard time with a LC*pro*+ photosynthesis system (ADC BioScientific, Hoddesdon, UK) at a temperature of 32 ± 3 °C, photosynthetically active radiation of 1300 ± 276 μmol photons m^–2^ s^–1^, using a flow rate of 335 ± 5 μmol s^–1^. The responses of berry growth, solute accumulation, and leaf photosynthetic rate to root pressurization were analyzed by one-way ANOVA.

## Results

**Fig. 1.**
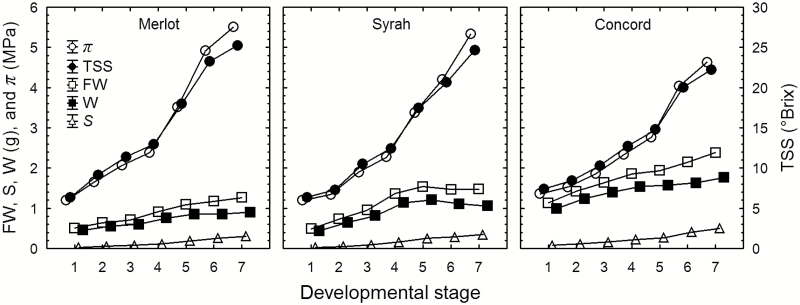
Changes in berry total soluble solids (TSS, closed circles), osmotic pressure (*π*, open circles), fresh weight (FW, open squares), water content (*W*, closed squares), and solute content per berry (*S*, open triangles) of Merlot, Syrah, and Concord grape berries at successive developmental stages (1=green hard; 2=green soft; 3=blush/pink; 4=red/purple; 5=blue; 6=ripe; 7=overripe). Values are means±SE where SE>symbol size (*n*=25).

**Fig. 2.**
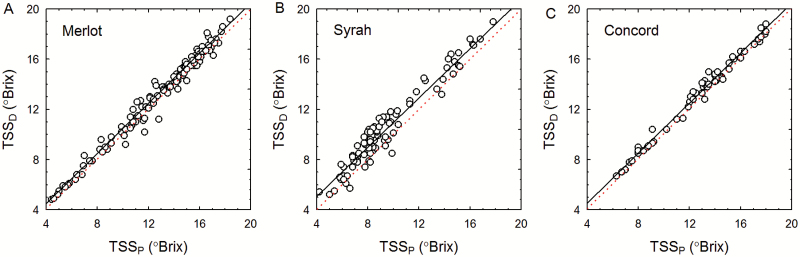
Association between total soluble solids (TSS) in the proximal (TSS_P_) and distal (TSS_D_) end sections of (A) Merlot, (B) Syrah, and (C) Concord grape berries. Regression analysis gave the following results for (A) *r*=0.99, *P*<0.001, y=0.40 + 0.99x, *n*=104; (B) *r*=0.98, *P*<0.001, y=1.07 + 0.99x, *n*=95; (C) *r*=0.98, *P*<0.001, y=0.50 + 0.99x, *n*=55. The dotted line in each panel is the 1:1 line with intercept=0. (This figure is available in colour at *JXB* online.)

Repeated measurements of the TSS and FW of Merlot, Syrah, and Concord berries sampled in the field at varying developmental stages showed that during ripening *S* per berry increased 8-, 11-, and 5-fold, respectively, while FW increased only 2-, 3-, and 2-fold, respectively (Fig. 1). These results show that solute accumulation outpaced berry weight gain markedly in all three genotypes. Across all ripening stages, TSS was consistently and significantly higher in the distal sections than in the middle and proximal sections of Merlot, Syrah, and Concord berries (*P*<0.001). The ΔTSS (intercept in Fig. 2) between the distal and proximal sections of Merlot, Syrah, and Concord berries was 0.40 ± 0.04, 1.07 ± 0.07, and 0.50 ± 0.05 °Brix, respectively.

**Fig. 3.**
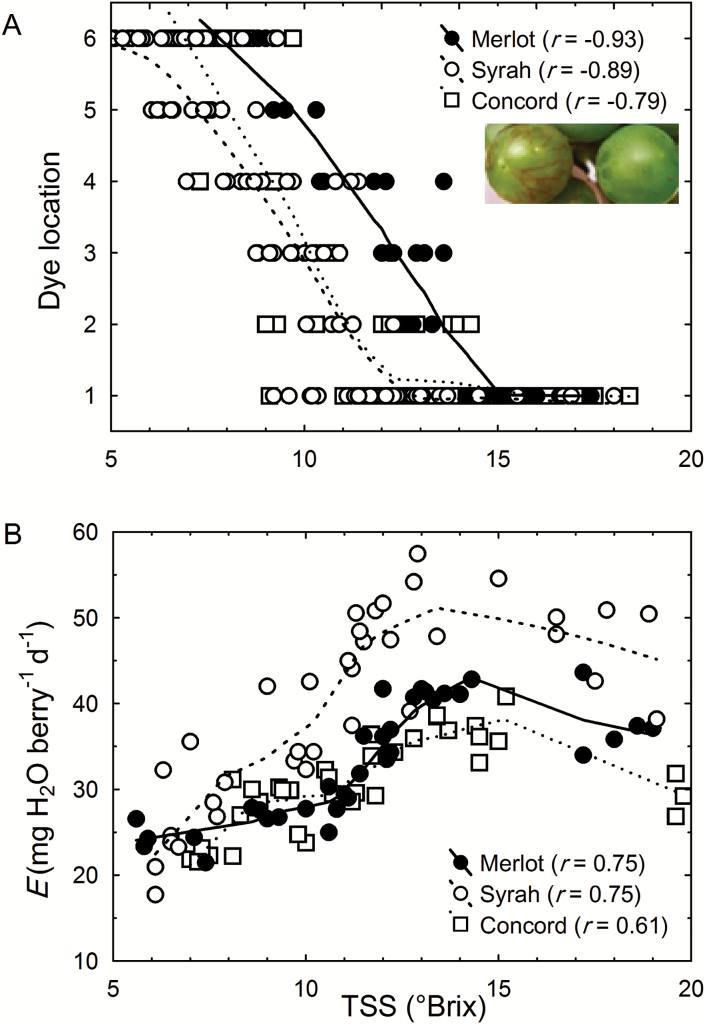
Associations between berry total soluble solids (TSS) and (A) xylem-mobile dye location inside berries and (B) transpiration rate (*E*) per berry of Merlot (closed circles), Syrah (open circles), and Concord (open squares) grapes. The extent of dye movement was quantified as 1 (dye in brush or proximal end only) to 6 (dye throughout berry vasculature). All *P*<0.001, *n*>50. Curves were fitted by the Lowess method. Inset in A shows extensive red-colored dye movement in a green hard berry (left) but not in an adjacent green soft berry (right) on the same Syrah cluster after 30 min of dye infusion.

**Fig. 4.**
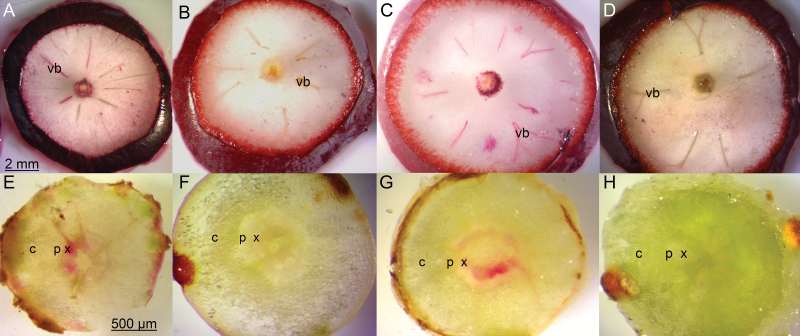
Cross-sections of Merlot (A, B, E, F) and Concord (C, D, G, H) berries and pedicels following infusion of basic fuchsin through the distal end of berries attached to plants with (B, D, F, H) and without (A, C, E, G) their roots pressurized. Without root pressurization, red-colored dye was present in the xylem of the berries (A, C) and pedicels (E, G). With pressurization, dye was absent in berries (B, D) and pedicels (F, H). Scale bars refer to their respective rows of panels. c, cortex; p, phloem; vb, vascular bundle; x, xylem.

When xylem-mobile dye was infused through the peduncle of Merlot, Syrah, and Concord fruit clusters, the extent of inward dye mobility into berries consistently decreased as TSS increased (Fig. 3A). Dye moved throughout the vascular bundles of green hard berries (<8 °Brix, *π* <1.3 MPa) within <2 h. By contrast, the dye remained confined to the brush region in blue-colored berries (>15 °Brix, *π* >2.7 MPa) even after 48 h of infusion. A striking difference in dye mobility was observed between adjacent berries on the same cluster immediately before and after the onset of ripening but before any color change in the skin: the dye was easily visible in green hard berries but not in green soft berries (inset in Fig. 3A). In contrast to the inverse correlation between TSS and dye location after 24 h of infusion (Fig. 3A), the transpiration rate per berry (*E*) increased 2- to 3-fold between 5 and 15 °Brix and then began to decline slightly (Fig. 3B). The temporary increase in *E* occurred while the berries doubled or tripled in size and changed from green hard to blue (Fig. 1). When dye was infused from the distal end of Merlot and Concord berries without root pressurization (control), it moved back through the xylem of berries (Fig. 4A, C) and the pedicels (Fig. 4E, G) regardless of ripeness level (12 to 22 °Brix). However, when the roots were pressurized, no dye movement was observed through the berry (Fig. 4B, D) or pedicel xylem (Fig. 4F, H). Dye movement was absent even when the applied pressure was as low as 0.1 MPa.

**Fig. 5.**
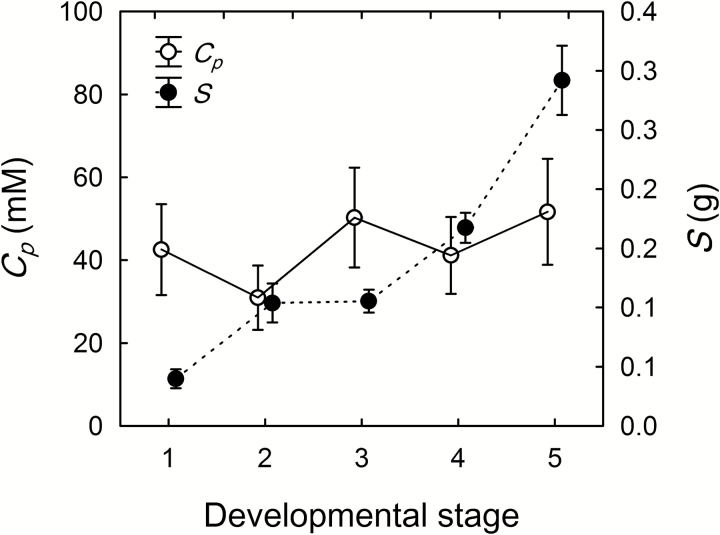
Changes in sucrose concentration (*C*_p_, open circles) in the phloem sap collected from grape berry pedicels, and solute content (*S*, closed circles) of the corresponding berries at successive developmental stages (1=green hard; 2=green soft; 3=blush/pink; 4=red/purple; 5=blue). Values for three *Vitis* genotypes (Merlot, Syrah, and Concord) were pooled because they did not differ significantly (*P*=0.31). Values are means±SE (*n*≥15, *P*=0.64 for *C*_*p*_, *P*<0.001 for *S*).

**Fig. 6.**
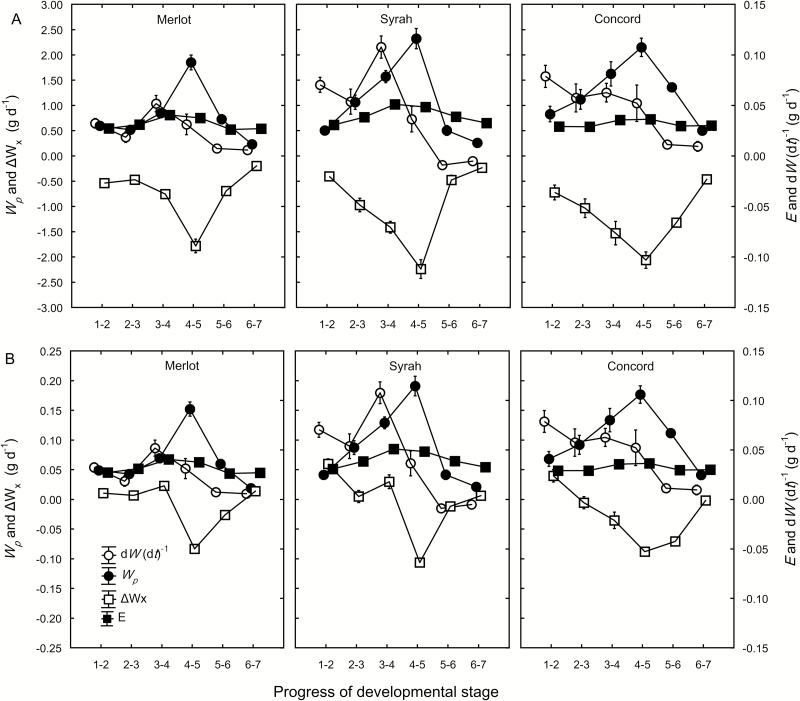
Rates of berry water accumulation [d*W* (d*t*)^–1^, open circles], phloem water inflow (*W*_p_, closed circles), net xylem flow (Δ*W*_x_, open squares), and berry transpiration (*E*, closed squares) of Merlot, Syrah, and Concord grapes between successive developmental stages (1=green hard; 2=green soft; 3=blush/pink; 4=red/purple; 5=blue; 6=ripe; 7=overripe). *W*_p_ and Δ*W*_x_ were estimated using a fruit growth model from measured *E*, berry weight, and solute concentration, and two different phloem sap sugar concentrations that were (A) measured in pedicel phloem sap or (B) the average for transport phloem reported in Jensen *et al.* (2013). Note the scale difference for *W*_p_ and Δ*W*_x_ compared with *E* and d*W* (d*t*)^–1^ in both A and B, and also the scale difference for the left y-axis between A and B. Values are means±SE where SE>symbol size (*n*=5, 10 berries per replicate).

In order to estimate the amount of phloem inflow to the berries, phloem sap was collected from individual pedicels and *C*_p_ was measured. No difference in *C*_p_ was found among the three genotypes (*P*=0.31). Despite a marked increase in *S* during development, there was no change in *C*_p_ (Fig. 5). Thus, the average measured *C*_p_ (46 ± 5.5 mM) was used in the fruit growth model. Overall, d*W* (d*t*)^–1^ and *E* were one order of magnitude lower than *W*_p_ and Δ*W*_x_ (Fig. 6A). In both Merlot and Syrah, d*W* (d*t*)^–1^ was highest between the blush/pink and red/purple stages (*P*<0.05). In Syrah only, d*W* (d*t*)^–1^ became negative in the ripe and overripe stages, consistent with the visible berry shrinkage in this genotype. In Concord, d*W* (d*t*)^–1^ was nearly steady from the green hard stage to the blue stage, and then decreased by 80% to the overripe stage (*P*<0.01). In order to meet the demand for solute accumulation (Fig. 1), *W*_p_ increased 3-, 5-, and 3-fold for Merlot, Syrah, and Concord, respectively, from the green hard stage (0.5–0.8 g d^–1^) to the blue stage (1.9–2.3 g d^–1^) (Fig. 6A). In overripe berries, *W*_p_ declined approximately 7-fold (to 0.3 g d^–1^ for Merlot and Syrah) or 4-fold (to 0.6 g d^–1^ for Concord) compared with its peak value. The average *W*_p_ for Syrah and Concord berries was 1.6-fold higher than that for Merlot (*P*=0.001). The combined water demand for berry growth and transpiration [d*W* (d*t*)^–1^+*E*] was only 3–20% of *W*_p_. Therefore, Δ*W*_*x*_ was negative (–0.2 to –2.2 g d^–1^), indicating xylem backflow, throughout berry ripening (Fig. 6A), and in a similar range as *W*_p_. The developmental profile of Δ*W*_x_ mirrored that of *W*_p_: Δ*W*_x_ became more negative during early ripening and less negative from the blue to the overripe stage. When the much higher *C*_p_ value of 532 mM from Jensen *et al.* (2013) was used in the model, *W*_p_ followed the same developmental pattern as with *C*_*p*_=46 mM but decreased 12-fold (Fig. 6B). Under this conservative scenario, d*W* (d*t*)^–1^+*E* was 105–244% of *W*_p_ from the green hard to the red/purple stage and from the ripe to the overripe stage in Merlot and Syrah, and from the green hard to the green soft stage in Concord. Therefore, Δ*W*_x_ was positive (0.005–0.06 g d^–1^), indicating xylem inflow, during these stages (Fig. 6B). From the blue to the ripe stage in Merlot and Syrah, and from the green soft to the overripe stage in Concord, d*W* (d*t*)^–1^+*E* was 37–96% of *W*_p_; thus, Δ*W*_*x*_ was negative (–0.002 to –0.1 g d^–1^) (Fig. 6B).

**Fig. 7.**
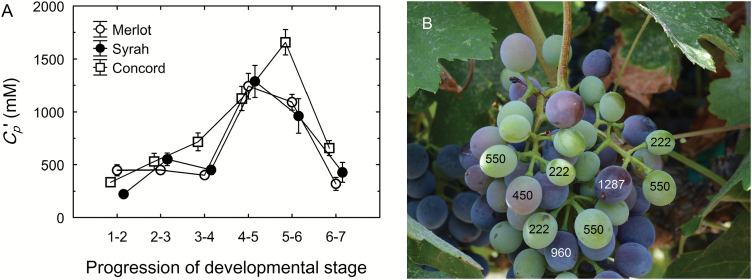
Hypothetical sucrose concentration (*C*_p_’) of the pedicel phloem sap as estimated from the fruit growth model for the scenario that all phloem-imported water is used for berry volume growth and transpiration (i.e. no net xylem flow between berry and pedicel). (A) Changes in *C*_p_’ of Merlot (open circles), Syrah (closed circles), and Concord (open squares) grapes throughout berry ripening (1=green hard; 2=green soft; 3=blush/pink; 4=red/purple; 5=blue; 6=ripe; 7=overripe). Values are means±SE where SE>symbol size (*n*=5). (B) Example of asynchronous ripening of berries on a single Syrah cluster at veraison. Numbers on the berries indicate the estimated *C*_p_’ for those berries. Note the variation in developmental stages of neighboring berries and the large discrepancy in *C*_p_’ for pedicels connected at the same rachis branching point.

For the hypothetical condition of Δ*W*_x_=0, the predicted *C*_p_’ was 5- to 36-fold higher than the measured *C*_p_. At the beginning of ripening, *C*_p_’ was 447 mM for Merlot, 222 mM for Syrah, and 335 mM for Concord (Fig. 7A). At the transition to the blue or ripe stage, *C*_p_’ reached its peak value (>1000 mM), representing a 3-, 6-, and 5-fold increase for Merlot, Syrah, and Concord, respectively (*P*<0.001). Thereafter *C*_p_’ decreased by 74%, 83%, and 60% for Merlot, Syrah, and Concord, respectively, in overripe berries. Due to the natural asynchrony of berry ripening, there may be a more than 3-fold difference in TSS among berries on the same fruit cluster at veraison, even though their pedicels may be connected at the same rachis branching point (Figs. 1 and 7B). At Δ*W*_x_=0, this would translate to a 3- to 6-fold difference in *C*_p_’ in the pedicels serving these berries (Fig. 7B). During late ripening, berries on the same cluster may vary from blue to overripe, which would still translate to a 2- to 3-fold difference in *C*_p_’ among individual pedicels.

**Fig. 8.**
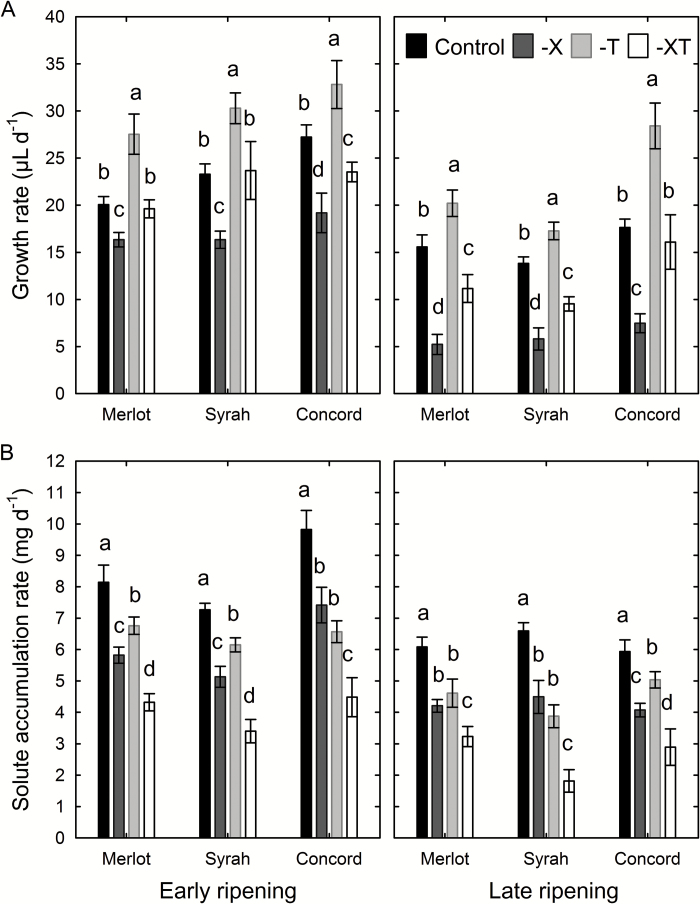
Effects of restricted xylem flow or berry transpiration on ripening of Merlot, Syrah, and Concord grapes. (A) Growth rate and (B) solute accumulation rate of berries from control clusters, clusters with restricted xylem flow (-X), restricted transpiration (-T), and the combination of both treatments (-XT). Treatments were in place for 14 d during both early and late ripening. Values are means±SE. Different letters indicate significant differences between means within genotypes at either early or late ripening (*n*=10, *P*<0.05).

The rates of volume growth and solute accumulation in berries from clusters with restricted xylem flow and/or transpiration were estimated and compared with berries from control clusters. Although the absolute rates of both berry growth and solute accumulation were higher during early ripening than during late ripening (*P*<0.05), the treatment effects were consistent across the two periods. Restricting berry transpiration enhanced berry growth, while xylem removal decreased berry growth, regardless of genotype (Fig. 8A). By contrast, restriction of xylem flow and/or transpiration always led to lower rates of solute accumulation (Fig. 8B). This effect was greatest when both xylem flow and transpiration were restricted. The lower solute accumulation rate was not due to a dilution effect, since what was compared was the change in the total amount per berry rather than the concentration. In addition, following the pre-veraison treatment application, slower progression of color change in treated clusters relative to control clusters was observed in all genotypes (Supplementary Fig. S3). Some berries split during the experiment. On average, berries of control clusters had a splitting frequency of 3%; restricting berry transpiration increased splitting nearly 6-fold in all genotypes (*P*<0.05), whereas restricting both xylem flow and transpiration increased splitting 2-fold in Concord and Syrah only (*P*<0.05). No difference in splitting frequency was found associated with treatment time (*P*=0.11).

**Fig. 9.**
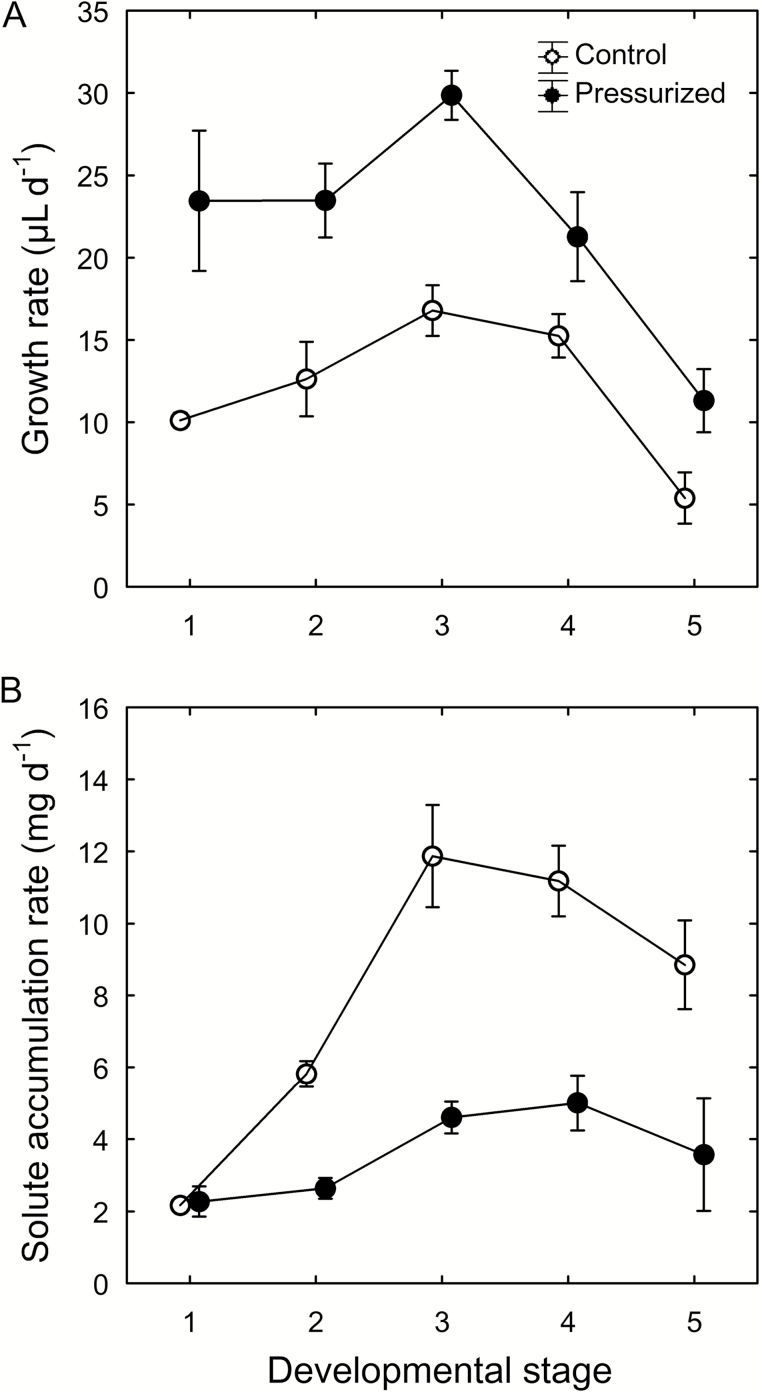
Effects of root pressurization on ripening of Syrah grapes. (A) Growth rate and (B) solute accumulation rate of berries from control (open circles) and pressurized (closed circles) plants. Berries were categorized based on their developmental stage at the beginning of the 3 d experiment (1=green hard; 2=green soft; 3=blush/pink; 4=red/purple; 5=blue). All stages of berries were present on each plant. Values are means±SE, where SE>symbol size (*n*=5).

Pressurizing the roots of potted vines increased berry growth rates by 80% (Fig. 9A) but decreased absolute solute accumulation rates by 49% across developmental stages compared with control berries (Fig. 9B). Generally, control berries advanced to the next stage (e.g. from green hard to green soft, or from red/purple to blue) over the 3 d of this experiment, whereas no progression was observed for most berries on root-pressurized plants. The difference in solute accumulation rate between pressurized and control berries increased during and after berry softening, while the difference in growth rate decreased after berry softening. Root pressurization did not alter leaf photosynthetic rate (4.4 ± 0.7 and 4.0 ± 0.7 μmol CO_2_ m^–2^ s^–1^ for control and pressurized vines, respectively; *P*=0.74), indicating that slower solute accumulation in the sink organs was not caused by reduced source activity.

## Discussion

At the onset of grape ripening, a combination of three processes may be expected to generate an increase in the driving force (∆*P*_x_) for xylem inflow into the berries: (i) pericarp expansion (Fig. 1); (ii) increase in vacuolar osmotic pressure (*π*) due to sugar accumulation (Fig. 1); and (iii) transient increase in berry transpiration (Fig. 3B). However, xylem-mobile dye movement into the berries declined at precisely the same time (Fig. 3A), even though whole berry *k*_h_ did not change (Choat *et al.*, 2009). The present study demonstrated that the decline in xylem inflow was related to changes in the direction of ∆*P*_x_. Altering ∆*P*_x_ halted the outward movement of xylem-mobile dye from the berries to the shoot (Fig. 4) and restored inward dye mobility into ripening berries (Bondada *et al.*, 2005; Chatelet *et al.*, 2008*a*; Keller *et al.*, 2015). Measurements of pedicel phloem sap sugar concentration (Fig. 5) and model calculations (Figs 6 and 7) support the idea that phloem inflow may exceed the berries’ water demand for growth and transpiration during ripening (Keller *et al.*, 2015). Under our experimental conditions and using the measured phloem sap sugar concentration (*C*_p_=46 mM), only a fraction of phloem-derived water (*W*_p_) was used for berry growth [d*W* (d*t*)^–1^)] and transpiration (*E*). The remaining *W*_p_ was likely discharged via the xylem. Even when we used the 12-fold higher *C*_p_ (532 mM) that is deemed near optimal for sugar transport (Jensen *et al.*, 2013), *W*_p_ still led to xylem backflow during some or most of the ripening phase, depending on genotype (Fig. 6B). When the xylem was disrupted, berry ripening (i.e. solute accumulation and color change) was impaired (Figs 8B, 9B; Supplementary Fig. S3). A similar effect was found when berry transpiration was partially blocked (Fig. 8B). These results provide further evidence that sugar accumulation in ripening grape berries requires the discharging of excess phloem-derived water, either across the berry skin (transpiration) or via the xylem (backflow).

Despite their presumably identical upstream *P*_x_ and *k*_h_ in the cluster peduncle, berries at different developmental stages on the same cluster varied in their inward dye mobility (Fig. 3A; Keller *et al.*, 2006). Since pedicel and berry *k*_h_ ostensibly do not change at the onset of ripening (Choat *et al.*, 2009; Knipfer *et al.*, 2015), the developmental decline in xylem flow may be explained only by changes in *P*_x_. When whole-plant *P*_x_ was increased to values at or above the balancing pressure, outward movement of xylem-mobile dye from berries was stopped (Fig. 4), whereas inward dye movement was restored (Bondada *et al.*, 2005; Chatelet *et al.*, 2008*a*; Keller *et al.*, 2015). This suggests that xylem flow in ripening berries remains responsive to *P*_x_. Despite a temporary increase in *E* during early ripening (Fig. 3B), which was mostly attributable to an increase in surface area due to berry growth (Fig. 1; Zhang and Keller, 2015), the strong opposing change in inward dye mobility (Fig. 3A) indicates that berry transpiration ceases to function as the main driving force for xylem flow. The case might be different for other fruits, such as kiwifruit berries, whose *E* may be higher than the rates of phloem inflow and fruit growth (Clearwater *et al.*, 2012; Morandi *et al.*, 2010).

The higher TSS in the distal section than the proximal section of ripening berries (Fig. 2) suggests that sugar accumulation may start in the distal end (see also Castellarin *et al.*, 2011) and then proceed at similar rates across a berry. Phloem unloading shifts from symplastic to apoplastic at the beginning of grape berry ripening (Zhang *et al.*, 2006). The corresponding increase in *π*_a_ (Keller *et al.*, 2015; Wada *et al.*, 2009) facilitates water efflux from the phloem (Patrick, 1997). If this efflux occurs first in the distal end of a berry, an increase in *P*_a_ there would be expected. Although pressure acts in all directions, it should be transmitted preferentially down the pathway with the least resistance, that is, the xylem conduits. Therefore, initiation of apoplastic phloem unloading in the distal end of a grape berry could lead to a Δ*P*_x_ pointing from the distal end toward the pedicel, similar to what has been reported for ripening fruit of prickly pear cactus (*Opuntia ficus-indica* L.; Nobel *et al.*, 1994).

Our measurements of *C*_p_ and the berry growth model support the notion of a Δ*P*_x_ from berry to pedicel. The *C*_p_ found here (46 ± 5.5 mM) was similar to reported values for pedicels of grape berries and other fruits (15–100 mM; Najla *et al.*, 2010; Zhang *et al.*, 2004, 2006), but significantly below the range of values (126–1472 mM) reported for transport phloem in other species (Jensen *et al.*, 2013). Because a *C*_p_≈50 mM might be viewed critically by phloem specialists, we used measurements of berry growth, solute accumulation, and transpiration to model berry vascular flows with both our measured *C*_p_ and the average *C*_p_ (532 mM) of all species included in Jensen *et al.* (2013). The model showed that, although increasing *C*_p_ 12-fold reduced *W*_p_ proportionately, the developmental pattern was similar for both scenarios (Fig. 6). Importantly, *W*_p_ increased several-fold during early grape ripening, confirming empirical results obtained using drought-stressed plants (Keller *et al.*, 2015). With *C*_p_=46 mM, only a small portion (≤20%) of the phloem-derived water was used to meet the demand for berry growth and transpiration, while the rest was recycled through the xylem (Fig. 6A). Following Münch’s (1930) original idea, similar observations have been reported for the fruit of cowpea (*Vigna unguiculata* L.; Pate *et al.*, 1985), prickly pear cactus (Nobel *et al.*, 1994), and mango (Higuchi and Sakuratani, 2006), while xylem backflow from kiwifruit berries was observed only in drought-stressed plants (Clearwater *et al.*, 2012). With *C*_p_=532 mM, 44–244% of *W*_p_ was necessary to meet the growth and transpiration demand, depending on the developmental stage and genotype. Even under this conservative scenario, however, there was still xylem backflow when sugar demand was at its peak in Merlot and Syrah berries, and throughout most of the ripening period in Concord berries (Fig. 6B). However, the prediction of xylem inflow during late ripening conflicts with results from dye infusion experiments (Fig. 3A; Keller *et al.*, 2006).

To test how *C*_p_ would have to change if there was no net xylem flow between a berry and its pedicel, we constrained our model parameters such that Δ*W*_x_=0. The predicted *C*_p_’ for this hypothetical scenario changed up to 6-fold during ripening in accordance with the berries’ sugar demand (Fig. 7). However, it seems unlikely that asynchronously developing berries on the same fruit cluster will have different sugar concentrations in their pedicel phloem (Fig. 7B), considering that multiple pedicels may be fed by the same upstream phloem sap. It seems far more likely that the upstream *C*_p_ is the same for these berries (Fig. 5), but that each berry, by lowering the sink phloem pressure, adjusts *W*_p_ to match its sink demand according to its developmental stage (Fig. 6). This conclusion is further supported by the striking differences in the movement of xylem-mobile dye into berries of differing developmental stages on the same cluster (Fig. 3A) and by the berry transpiration and xylem manipulation experiments (Figs 8 and 9).

Restricting the discharge of surplus phloem water compromised berry solute accumulation (Figs 8B and 9B) and color change (Supplementary Fig. S3), indicating that phloem inflow was reduced. At first glance, the lower berry growth rate following xylem removal (Fig. 8A) and the higher growth rate under root pressurization (Fig. 9A) seem to suggest that these treatments simply altered xylem inflow. While this may have been partly the case, especially for the pre-veraison treatment, this interpretation cannot explain the lower absolute solute accumulation rate (i.e. change in solute amounts per berry, rather than solute concentrations) in both treatments, which must have been associated with reduced phloem inflow. Despite our precautions, it was possible that xylem drilling might have inflicted minor damage to the phloem at the shoot/peduncle junction and/or resulted in temporary callose formation in the phloem (e.g. Furch *et al.*, 2007; Jaffe *et al.*, 1985). This could have contributed to the lower rates of berry growth and solute accumulation. This interpretation would also explain the finding that the berry splitting frequency in the combined treatment (-XT) was intermediate between that in the -X and -T treatments. However, the fact that solutes accumulated in the berries of all -X and -XT clusters, albeit at lower rates than in the control clusters, demonstrates that xylem removal decreased but did not eliminate phloem flow through the peduncle. Consistent with this conclusion, peduncle girdling (i.e. phloem removal) but not xylem removal prevented berry growth and solute accumulation (Hall *et al.*, 2011; Keller *et al.*, 2015), and restricting berry transpiration reduced solute accumulation (Rebucci *et al.*, 1997). Moreover, root pressurization, which curtailed outward dye movement (Fig. 4), also decreased berry solute accumulation (Fig. 9B) and color change. It is not known whether root pressurization may affect phloem transport or overall carbon partitioning directly; however, no effect on leaf photosynthesis was found in the present study. Taken together, these results strongly suggest that both berry transpiration and xylem backflow serve as water discharge pathways to facilitate phloem unloading and sugar accumulation during grape ripening. Perturbation of one or both of these pathways may hamper the release of phloem pressure at the sink end (see also Münch, 1930; Patrick, 1997), which would reduce the rate of phloem inflow and hence sugar delivery. The magnitude and direction of xylem flow may be variable, depending on *C*_p_ and berry sugar demand and transpiration, and the ability to recycle surplus phloem water via the xylem may be especially important during periods of rapid sugar accumulation and under environmental conditions that limit berry transpiration (i.e. low vapor pressure deficit; Zhang and Keller, 2015).

## Supplementary data

Supplementary data are available at *JXB* online.

Fig. S1. Association between total soluble solids and osmotic pressure in ripening Merlot and Concord grape berries.

Fig. S2. Longitudinal section of a pre-veraison grape berry infused with the xylem-mobile dye basic fuchsin to visualize axial and peripheral vascular bundles.

Fig. S3. Images of Syrah grape clusters that were either untreated or had their peduncle xylem destroyed to restrict xylem flow and/or were treated with antitranspirant to restrict transpiration.

## Supplementary Material

supplementary_figure_S1_S3

## Abbreviations:

*π*_a_: apoplast osmotic pressure
*P*_a_: apoplast pressure
E: berry transpiration rate
*k*_h_: hydraulic conductance
P: hydrostatic pressure
Δ*P*_x_: hydrostatic pressure gradient in the xylem
π: osmotic pressure
TSS: total soluble solids
*P*_x_: xylem pressure.
