# Supplementary material for: Discharge of surplus phloem water may be required for normal grape ripening
Source: J Exp Bot. 2017 Jan 12;68(3):585–95. doi: 10.1093/jxb/erw476 (PMC5444433; doi:10.1093/jxb/erw476)
Supplement: supplementary_figure_S1_S3 [file erw476_suppl_supplementary_figure_S1_S3.pdf]

Discharge of surplus phloem water may be required for normal grape ripening

Yun Zhang, Markus Keller

Supplementary figure legends:

Supplementary Figure S1. Association between total soluble solids (TSS) and osmotic pressure (π) in ripening Merlot and Concord grape berries. The dotted red line is the linear fit.

Supplementary Figure S2. Longitudinal section of a pre-veraison grape berry infused with the xylem-mobile dye basic fuchsin to visualize axial and peripheral vascular bundles (arrows). Three sections for solute measurements are shown. Ovals indicate seed locations.

Supplementary Figure S3. Images of Syrah grape clusters that were untreated (Control), had their peduncle xylem destroyed to restrict xylem flow (-X), were treated with antitranspirant to restrict transpiration (-T), or had both treatments applied together (-XT), 14 days after the pre-veraison treatment application.

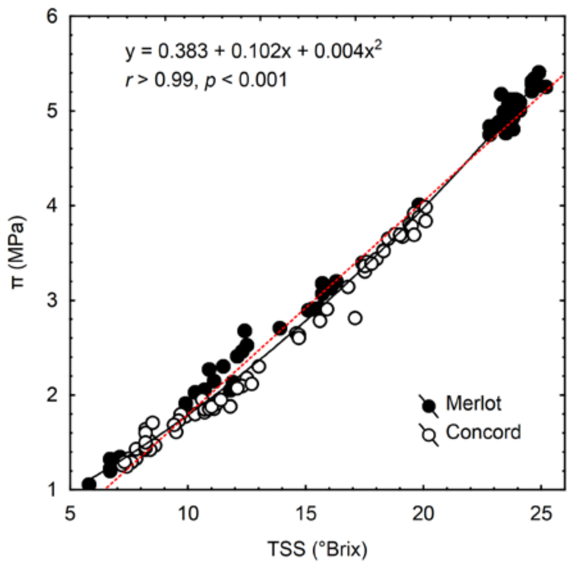

Supplementary Figure S1. Association between total soluble solids (TSS) and osmotic pressure (π) in ripening Merlot and Concord grape berries. The dotted red line is the linear fit.

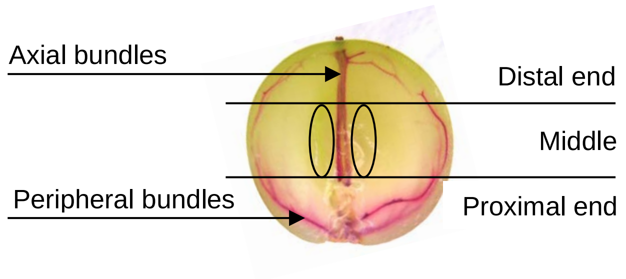

Supplementary Figure S2. Longitudinal section of a pre-veraison grape berry infused with the xylem-mobile dye basic fuchsin to visualize axial and peripheral vascular bundles (arrows). Three sections for solute measurements are shown. Ovals indicate seed locations.

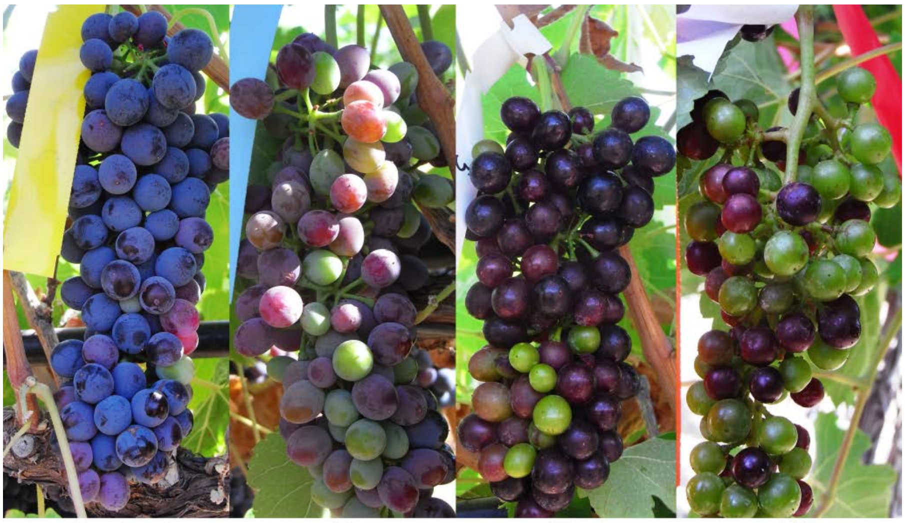

Control -X -T -XT

Supplementary Figure S3. Images of Syrah grape clusters that were untreated (Control), had their peduncle xylem destroyed to restrict xylem flow (-X), were treated with antitranspirant to restrict transpiration (-T), or had both treatments applied together (-XT), 14 days after the pre-veraison treatment application.
